# Validity of predictive equations to estimate RMR in females with varying BMI

**DOI:** 10.1017/jns.2020.11

**Published:** 2020-05-26

**Authors:** George Thom, Konstantinos Gerasimidis, Eleni Rizou, Hani Alfheeaid, Nick Barwell, Eirini Manthou, Sadia Fatima, Jason M. R. Gill, Michael E. J. Lean, Dalia Malkova

**Affiliations:** 1 Human Nutrition, School of Medicine, College of Medical, Veterinary and Life Sciences, University of Glasgow, New Lister Building, Glasgow Royal Infirmary, Glasgow G31 2ER, UK; 2 Qassim University, Buraydah City, P. C. 51452, Saudi Arabia; 3 BHF Glasgow Cardiovascular Research Centre, Institute of Cardiovascular and Medical Sciences, College of Medical, Veterinary and Life Sciences, University of Glasgow, Glasgow G12 8TA, UK

**Keywords:** RMR, Prediction equations, Mifflin–St Jeor equations, Henry equations, Schofield equations, Harris–Benedict equations, Owen equations

## Abstract

Estimation of RMR using prediction equations is the basis for calculating energy requirements. In the present study, RMR was predicted by Harris–Benedict, Schofield, Henry, Mifflin–St Jeor and Owen equations and measured by indirect calorimetry in 125 healthy adult women of varying BMI (17–44 kg/m^2^). Agreement between methods was assessed by Bland–Altman analyses and each equation was assessed for accuracy by calculating the percentage of individuals predicted within ± 10 % of measured RMR. Slopes and intercepts of bias as a function of average RMR (mean of predicted and measured RMR) were calculated by regression analyses. Predictors of equation bias were investigated using univariate and multivariate linear regression. At group level, bias (the difference between predicted and measured RMR) was not different from zero only for Mifflin–St Jeor (0 (sd 153) kcal/d (0 (sd 640) kJ/d)) and Henry (8 (sd 163) kcal/d (33 (sd 682) kJ/d)) equations. Mifflin–St Jeor and Henry equations were most accurate at the individual level and predicted RMR within 10 % of measured RMR in 71 and 66 % of participants, respectively. For all equations, limits of agreement were wide, slopes of bias were negative, and intercepts of bias were positive and significantly (*P* < 0⋅05) different from zero. Increasing age, height and BMI were associated with underestimation of RMR, but collectively these variables explained only 15 % of the variance in estimation bias. Overall accuracy of equations for prediction of RMR is low at the individual level, particularly in women with low and high RMR. The Mifflin–St Jeor equation was the most accurate for this dataset, but prediction errors were still observed in about one-third of participants.

Estimation of RMR using prediction equations is a fundamental part of clinical dietetic practice and is the basis for estimating daily energy requirements using the factorial method^(1)^. Despite known limitations, prediction equations offer a practical alternative to measuring RMR by indirect calorimetry since energy requirements can be calculated using routinely available measures such as weight, age, sex and height. Expense of indirect calorimetry equipment, requirement for trained personnel, and the time-consuming nature of RMR measurement prevent indirect calorimetry being routinely available in dietetic settings^(2)^, though it is frequently used in research studies.

RMR accounts for 60–70 % of total daily energy expenditure and fat-free mass (FFM) is the primary determinant of energy expended at rest^(3)^. FFM consists of multiple organs and tissues with varying metabolic rate^(4)^. Despite comprising <6 % of total body weight, organs are the major drivers of RMR, with the brain, heart, kidneys and liver collectively accounting for 60–80 % and although skeletal muscle represents a greater proportion of FFM, its metabolic activity is lower^(4,5)^. Since accurate measurement of FFM is not routinely available, RMR equations usually rely on body weight as the dominant predictor, and also consider age, sex and sometimes height^(2)^. There are many factors influencing RMR and there is considerable individual variation even when adjusting for FFM, age and sex^(3)^.

Numerous published prediction equations are available to estimate RMR, with 248 different formulas being identified within the literature^(6)^. The equations of Schofield^(7)^, Henry^(8)^, Mifflin–St Jeor^(9)^, Harris–Benedict^(10)^ and Owen^(11,12)^ are all widely used. There have been several studies evaluating prediction equation accuracy over the years, including investigations in European^(13–17)^ and US populations^(13,18–20)^, but regular revalidation and updating of recommendations remain important due to changing demographics of modern populations, as well as changes in body composition. More specifically, in recent years the European Food Safety Authority recommended the Henry equation for predicting RMR in all European Union countries^(21)^, and it is recommended for use by UK dietitians^(22)^, yet its predictive accuracy still needs to be established within the current population^(23)^, particularly within studies across BMI categories. The purpose of the present study was to evaluate the validity of several RMR prediction equations, by comparing RMR measured by indirect calorimetry with RMR predicted by equation, in a group of healthy women with varying BMI.

## Methods

### Participants

The present study is a secondary analysis of data collected from healthy adult women across energy balance research studies performed in the Human Nutrition Unit at the University of Glasgow between 2008 and 2017^(24–29)^. Participants (*n* 125; aged 20–57 years; BMI 17–44 kg/m^2^) were sedentary, non-smokers, not pregnant, not taking medication which could affect RMR and had maintained stable body weight for at least 2 months prior to study enrolment. They were asked to avoid exercise in the 24 h prior to testing. Only the baseline RMR values measured prior to participation in the interventions were considered for the present study. Four studies were approved by the College of Medical, Veterinary and Life Sciences of the University of Glasgow Ethics Committee^(24–28)^, and the other by the West of Scotland NHS Ethics Committee^(29)^. Studies were conducted in accordance with the Declaration of Helsinki, and all participants recruited provided written informed consent.

### Data collection

Participants reported to the metabolic investigation unit following a 12 h overnight fast. Height was measured to the nearest 0⋅1 cm using a stadiometer (Seca). Body weight was determined by bioelectrical impedance scales (TBF-300; Tanita), and values obtained were incorporated within prediction equations for estimation of RMR. Participants rested for 20 min prior to RMR measurement, which took place in a thermoneutral environment between 07.00 and 10.00 hours to ensure that fasting periods were not extended for too long during daytime hours. Indirect calorimetry was conducted using a calibrated open-circuit ventilated hood system, Oxycon Pro (Jaeger GmbH) or Deltatrac Metabolic Monitor (Datex Engstrom). RMR was obtained in sixty-six subjects using Deltatrac and fifty-nine subjects using Oxycon Pro. Both devices were calibrated prior to the measurements and alcohol-burning validation tests were conducted weekly on Oxycon Pro during testing periods. The measurements involved recording the rate of oxygen consumption (V˙O_2_) and carbon dioxide production (V˙CO_2_) every 30 s for the duration of the 20-min test period with participants lying in a supine position whilst fully awake. To ensure that steady-state conditions were obtained^(30)^, data from the first 5 min were discarded and V˙O_2_ and V˙CO_2_ measured during the following 15-min period were required to have a CV ≤10 % to be accepted for the calculation of RMR, which was achieved using the following indirect calorimetry equations, with reference to Frayn^(31)^:

Rate of fat oxidation (g/min) = (VO_2_, litres/min – VCO_2_, litres/min)/0⋅57

Rate of carbohydrate oxidation (g/min) = (1⋅4 × VCO_2_, litres/min – VO_2_, litres/min)/0⋅3

RMR (kcal/min) = (rate of fat oxidation × 9 kcal) + (rate of carbohydrate oxidation × 4 kcal)

RMR (kcal/24 h) = RMR (kcal/min) × 1440 (min)

(To convert kcal to kJ, multiply by 4·184.)

### Prediction equations

Of the many prediction equations that have been published in the literature, those of Harris–Benedict, Henry, Mifflin–St Jeor, Owen and Schofield are amongst the most widely used, either currently or in the recent past. Therefore, these equations were used in the present study to predict RMR and compared against measured RMR by indirect calorimetry. The RMR equations, and the populations from which they were derived are described in Table 1.

**Table 1.** Equations for predicting RMR in kcal/d*

Equation	Reference population	Prediction equation
Harris–Benedict^(10)^	*n* 239 (136 males and 103 females)	Female: 665⋅09 + 9⋅56 × weight + 1⋅84 × height – 4⋅67 × age Male: 66⋅47 + 13⋅75 × weight + 5⋅0 × height – 6⋅75 × age
Henry^(8)^	*n* 10 552 (5794 males and 4702 females)	Female (age between 18 and 30 years): 13⋅1 × weight + 558 Female (age between 30 and 60 years): 9⋅74 × weight + 694 Male (age between 18 and 30 years): 16⋅0 × weight + 545 Male (age between 30 and 60 years): 14⋅2 × weight + 593
Mifflin–St Jeor^(9)^	*n* 498 (251 males and 248 females)	Female: 9⋅99 × weight + 6⋅25 × height – 4⋅92 × age – 161 Male: 9⋅99 × weight + 6⋅25 × height – 4⋅92 × age + 5
Owen^(11,12)^	*n* 104 (60 males and 44 females)	Female: 795 + 7⋅18 × weight Male: 879 + 10⋅2 × weight
Schofield^(7)^	*n* 7173 males and females	Female (age between 18 and 30 years): 14⋅818 × weight + 486⋅6 Female (age between 30 and 60 years): 8⋅126 × weight + 845⋅6 Male (age between 18 and 30 years): 15⋅057 × weight + 692⋅2 Male (age between 30 and 60 years): 11⋅472 × weight + 873⋅1

* To convert kcal to kJ, multiply by 4·184.

### Statistical and data analysis

Accuracy of the five prediction equations was assessed at both group and individual levels. Results are presented as mean values and standard deviations. At group level, bias (predicted – measured RMR) was assessed by one-sample *t* test. The 95 % limits of agreement were calculated as the mean of the two values, ± 1⋅96 sd. Root-mean-square error was calculated to show the sample standard deviation of differences between predicted and measured RMR. Agreement between predicted and measured values was assessed by Bland–Altman analyses^(32)^. Slopes and intercepts of bias as a function of average RMR (mean of predicted and measured RMR) were calculated by regression analyses. At the individual level, RMR prediction was considered accurate if it was within ± 10 % of measured RMR, which is a standard approach for investigating accuracy of RMR prediction equations^(2)^ and in line with recommendations for conducting this type of research^(33)^. When predicted RMR was <90 and >110 % of measured RMR, it was considered as an underestimation and an overestimation, respectively. Accuracy was calculated for the whole group and separately by BMI category: underweight, healthy weight, overweight and obesity. Predictors (age, height and BMI) of the bias were investigated using univariate and multivariate regression analysis. Results were considered statistically significant if *P* < 0⋅05. Data were analysed in Minitab 17 and MedCalc 17.2.

## Results

Descriptive data of the study participants are shown in Table 2. Of the 125 participants included in the analysis, mean age was 30⋅7 (sd 8⋅8) years (range 20–57 years), body weight 70⋅8 (range 41⋅0–120⋅9) kg and BMI 26⋅1(sd 6⋅6) kg/m^2^ (range 16⋅8–44⋅4 kg/m^2^). Combined overweight and obesity prevalence was 58 %.

**Table 2.** Participant characteristics (Mean values and standard deviations)

	All		BMI < 18⋅5 kg/m^2^		BMI 18⋅5–24⋅9 kg/m^2^		BMI 25–29⋅9 kg/m^2^		BMI ≥30 kg/m^2^	
	Mean	sd	Mean	sd	Mean	sd	Mean	sd	Mean	sd
Subjects (*n*)	125		13		40		42		30	
Age (years)	30⋅7	8⋅8	23⋅5	3⋅1	27⋅1	5⋅1	31⋅8	7⋅6	37⋅0	10⋅9
Body weight (kg)	70⋅8	18⋅3	48⋅3	4⋅7	57⋅6	7⋅7	72⋅7	6⋅8	95⋅5	13⋅1
BMI (kg/m^2^)	26⋅1	6⋅6	17⋅7	0⋅4	21⋅0	2⋅0	27⋅1	1⋅4	35⋅3	4⋅4

Comparison of overall performance of the prediction equations is summarised in Table 3. At group level, the difference between predicted and measured RMR (bias) was not significant for the Mifflin–St Jeor and Henry equations, but for the Harris–Benedict, Schofield and Owen equations, predicted RMR values were significantly different from measured. The Mifflin–St Jeor and Henry equations were most accurate on an individual basis, and predicted RMR was within 10 % of measured values in 71 and 66 % of the participants, respectively. Accuracy for the Schofield, Harris–Benedict and Owen equations was 63, 61 and 59 %, respectively. Where loss of accuracy was observed, underpredictions were most common for the Owen equation, whereas overpredictions occurred more frequently for the other equations. Root-mean-square error data provided additional evidence of greater individual level accuracy for both the Mifflin–St Jeor and Henry equations.

**Table 3.** Evaluation of prediction equation accuracy in comparison with RMR measured by indirect calorimetry† (Mean values and standard deviations; limits of agreement (LOA); percentages)

	REE (kcal/d)		Bias (P-M)								Slope (kcal/d per 100 kcal/d)		Intercept (kcal/d)	
	Mean	sd	Mean	sd	95 % LOA (± 2 sd)	RMSE	Accurate predictions within ± 5 %‡ (%)	Accurate predictions within ± 10 %§ (%)	Underpredictionsǁ (%)	Overpredictions¶ (%)	Mean	sd	Mean	sd
RMR measured	1424	247												
Harris–Benedict	1502	162	78*	157	−229, 384	174	33⋅6	60⋅8	7⋅2	32⋅0	−4⋅6*	0⋅1	757*	88
Henry	1432	202	8	163	−311, 327	153	41⋅6	65⋅6	14⋅4	20⋅0	−0⋅2*	0⋅1	335*	96
Mifflin St–Jeor	1424	180	0	153	−300, 300	162	43⋅2	71⋅2	12⋅0	16⋅8	−3⋅5*	0⋅1	503*	86
Owen	1303	131	−121*	165	−443, 202	204	24⋅8	59⋅2	36⋅8	4⋅0	−6⋅8*	0⋅1	803*	76
Schofield	1466	211	42*	173	−297, 381	177	29⋅6	63⋅2	13⋅6	23⋅2	−1⋅8*	0⋅5	313*	83

REE, resting energy expenditure; P-M, predicted RMR minus measured RMR; RMSE, root-mean-square error calculated as √(Σ(actual RMR – predicted RMR)^2^)/*n*.

* Mean value was significantly different from zero (*P* < 0⋅05).

† To convert kcal to kJ, multiply by 4·184.

‡ Percentage of participants with predicted RMR within 5 % of measured RMR.

§ Percentage of participants with predicted RMR within 10 % of measured RMR.

ǁ Percentage of participants with predicted RMR being more than 10 % below measured RMR.

¶ Percentage of participants with predicted RMR being more than 10 % above measured RMR.

**Fig. 1.**
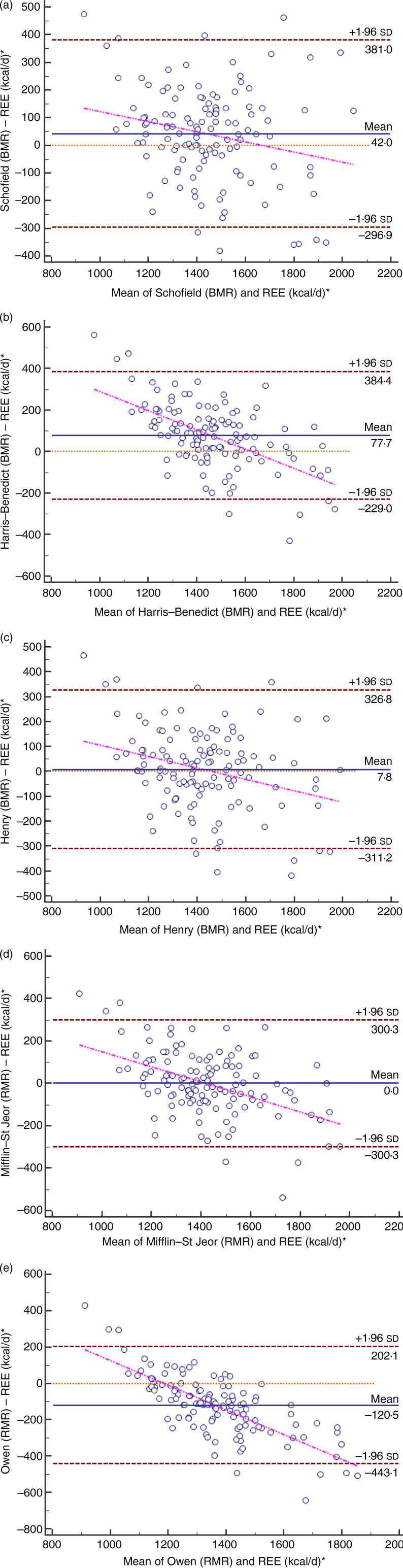
Bland–Altman plots of differences in RMR measured by indirect calorimetry and predicted using five different equations in 125 adult women. The solid line represents the mean difference (predicted – measured RMR). Upper and lower dashed lines represent the 95 % limits of agreement (±2 sd). The regression line indicates the difference between predicted and measured RMR, plotted against the mean. REE, resting energy expenditure. * To convert kcal to kJ, multiply by 4·184.

Fig. 1 shows the Bland–Altman plots which display differences in RMR measured by indirect calorimetry and predicted using the five equations. Limits of agreement were wide for all equations, ranging from an upper limit of +384 kcal/d (+1607 kJ/d) to a lower limit of −443 kcal/d (–1854 kJ/d) for the Harris–Benedict and Owen equations, respectively. For all Bland–Altman plots, slopes and intercepts, calculated from regression analyses between average RMR (mean of predicted and measured RMR) and the bias, were significantly different from zero. The slopes ranged between −6⋅8 (Owen) and −0⋅2 (Henry) and were negative for the equations.

**Fig. 2.**
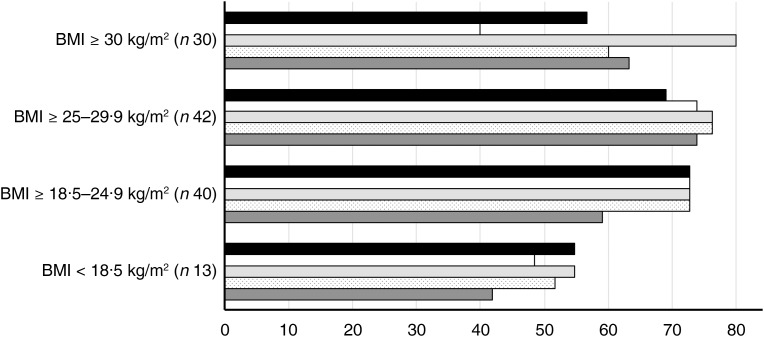
Percentage of adult women for whom RMR predicted by Schofield (■), Owen (□), Mifflin–St Jeor (
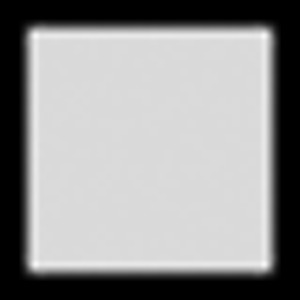
), Henry (
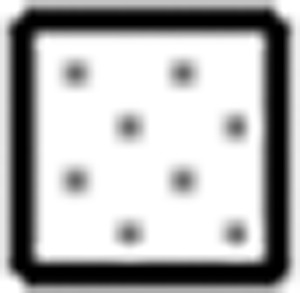
) and Harris–Benedict (
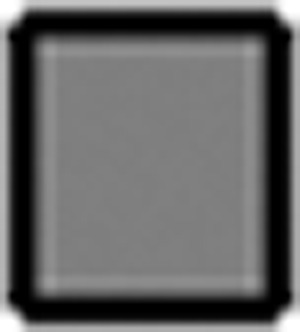
) equations was within ± 10 % of RMR measured by indirect calorimetry, according to BMI category (underweight, BMI <18 kg/m^2^; healthy weight, BMI ≥18⋅5–24⋅9 kg/m^2^; overweight, BMI ≥25–29⋅9 kg/m^2^, and obesity BMI ≥30 kg/m^2^).

For each equation, the percentage of accurate RMR predictions was assessed for each BMI category separately and results are presented in Fig. 2. In women who were underweight, estimation accuracy was lowest with ≤55 % of predictions being within 10 % of measured RMR. In women who were of healthy weight, prediction accuracy was better for the Schofield, Mifflin–St Jeor, Henry and Owen equations, with all equations achieving approximately 73 % accuracy, but the Harris–Benedict equation predicted RMR accurately in only 59 %. Prediction accuracy was higher for all equations in women who were overweight, ranging from 69 to 76 %. In women with obesity, the Mifflin–St Jeor equation was highly accurate and predicted RMR within 10 % of measured RMR in 80 % of cases. Accuracy rate was lower for all other equations, ranging from 40 to 63 %.

Table 4 shows predictors of the bias between estimated and measured RMR, based on multivariate linear regression analysis. Increasing age, height and BMI were significant predictors of RMR under- or overestimation, but this effect varied with all equations and collectively explained only a small proportion of the variance in estimation bias (maximum adjusted *R*^2^ 15 %). Thus, at least 85 % of the estimation bias was explained by other factors. In additional regression analyses, we found no difference in absolute and percentage prediction error between the Oxycon Pro and Deltatrac metabolic carts.

**Table 4.** Predictors of the difference between estimated and measured RMR based on univariate and multivariate linear regression†

	Harris–Benedict			Schofield			Owen			Mifflin–St Jeor			Henry		
Predictors	*R*^2^ (%)	β Coefficient % (kcal)	*P*	*R*^2^ (%)	β Coefficient % (kcal)	*P*	*R*^2^ (%)	β Coefficient % (kcal)	*P*	*R*^2^ (%)	β Coefficient % (kcal)	*P*	*R*^2^ (%)	β Coefficient % (kcal)	*P*
Univariate analysis															
Age	6⋅49	−0⋅4 (−4⋅91)	0⋅004**	4⋅89	−0⋅34 (−4⋅9)	0⋅013*	1⋅06	−0⋅142 (−2⋅59)	0⋅254	6⋅54	−0⋅355 (−4⋅91)	0⋅004**	3⋅23	−0⋅262 (−3⋅7)	0⋅025*
Height	4⋅74	−0⋅5 (−4⋅6)	0⋅015*	4⋅12	−0⋅454 (−4⋅95)	0⋅023*	9⋅84	−0⋅637 (−8⋅6)	<0⋅001***	0⋅16	−0⋅08 (0⋅14)	0⋅662	4⋅56	−0⋅458 (−5⋅16)	0⋅017*
BMI	6⋅4	−0⋅527 (−5⋅01)	0⋅004**	0⋅02	−0⋅028 (1⋅15)	0⋅881	6⋅19	−0⋅461 (−7⋅96)	0⋅005**	3⋅72	−0⋅359 (−4⋅21)	0⋅031*	0⋅01	−0⋅021 (0⋅93)	0⋅904
Multivariate analysis															
Age	N/A			N/A			N/A			*R*^2^ adj (%): 5⋅8			N/A		
Age and height	*R*^2^ adj (%): 10⋅54			*R*^2^ adj (%): 8⋅2			*R*^2^ adj (%): 15⋅3			N/A			N/A		
Age, height and BMI	N/A			N/A			N/A			N/A			*R*^2^ adj (%): 6⋅8		

N/A, not applicable; adj, adjusted.

* *P* < 0⋅05, ** *P* < 0⋅01, *** *P* < 0⋅001.

† To convert kcal to kJ, multiply by 4·184.

## Discussion

Different methods for determining RMR are not likely to agree exactly, but whether differences in accuracy are likely to affect patient management is an important clinical consideration. In the present study conducted on healthy females with varying BMI, there was considerable room for improvement in the performance of each of the prediction equations at individual-level assessment, though good agreement between measured and predicted RMR was observed at group level using the Mifflin–St Jeor and Henry equations. Although our data suggest that the most accurate prediction of RMR can be expected from using the Mifflin–St Jeor equation, we note that in nearly one-third of participants predicted RMR was outwith 10 % of measured RMR. The wide limits of agreement and slopes and intercepts being different from zero in the regression from Bland–Altman analysis for Mifflin–St Jeor, and indeed for all equations, suggest that there was an asymmetrical relationship in the bias and that RMR prediction at individual level should be interpreted with caution and considered only as a starting point for planning dietary interventions. The findings of the present study also imply that in healthy women at the extremes of BMI (underweight/obesity), and therefore with relatively low or high measured RMR, the validity of prediction equations in general is particularly poor, with the one exception being when applying the Mifflin–St Jeor equation to women with obesity. This is of concern given that underweight and obesity are clinical situations where equations are frequently used in practice. Despite relatively poor accuracy at the individual level, the equations investigated in the present study can be expected to operate well at population level to assist governments in planning and monitoring nutrition programmes.

In this dataset, the Mifflin–St Jeor equation performed best in women with healthy weight, overweight and obesity, predicting RMR accurately in 73, 76 and 80 % of the participants, respectively. Our findings that Mifflin–St Jeor is the most accurate prediction equation, especially for women with obesity, is in line with evidence from systematic reviews which recommend the Mifflin–St Jeor equation for use in individuals with overweight and obesity, and also report a similar number of inaccurate predictions^(2,33)^. These findings imply that the Mifflin–St Jeor equation should not necessarily be restricted to women with obesity, since it was also as accurate as other equations for estimating RMR in underweight, healthy-weight and overweight women. It is perhaps not surprising that the Mifflin–St Jeor equation provided the most consistent accuracy across BMI categories since it was developed in a large sample of individuals with healthy weight, overweight and obesity^(9)^. Although predicted RMR values do in part reflect the characteristics of the population they were derived from, and thus can lack specificity when applied to individuals different from the original population^(34,35)^, our data suggest that population-specific factors play a relatively minor role in influencing prediction of RMR, and the Mifflin–St Jeor equation has demonstrated the best accuracy in several other studies using differing populations^(13,20,36)^. The ± 10 % ‘rule’ for defining prediction accuracy is a pragmatic criterion which facilitates comparison between studies and is clinically relevant since chronic energy imbalances are likely to contribute to cases of malnutrition, or, conversely, to overweight and obesity. Due to the large number of confounding factors that exist, it is difficult to show clear links between adverse clinical outcomes and the selected cut-offs for under- and overfeeding (<90 or >110 % of energy requirements); however, respiratory and liver dysfunction, poor wound healing and infections, azotemia and renal failure, and total complication rate have been associated with under- and/or overfeeding in critically ill patients^(37–42)^. Furthermore, prescription of higher (or lower) than intended energy deficits to people seeking to correct or ameliorate overweight and obesity-related complications are likely to result in poorer adherence and weight-loss outcomes^(43)^.

The work of Schofield and colleagues^(7)^ was influential in re-establishing the use of RMR rather than reported food consumption as the basis for estimating energy requirements^(8)^, and was used to inform the development of dietary reference values for children and adults^(44)^. The Schofield equations were widely used by dietitians in clinical practice^(45)^ until relatively recently, but key advisory groups now recommend using the Henry equation, as the older Schofield equation overestimates RMR^(21–23)^. The newer Henry equation addressed some of the limitations of the Schofield equation by including data collected on greater numbers of people from tropical climates, and a large cohort of young, physically active Italian subjects were excluded who were found to have higher BMR per kg/body weight than other Caucasian groups^(8)^. We found no difference between RMR measured by indirect calorimetry and predicted RMR using the Henry equation at group level and 66 % of predictions were within 10 % of measured RMR. Thus, the Henry equation predicts RMR more accurately than the Schofield, Harris–Benedict and Owen equations at group and individual levels, though overall the Mifflin–St Jeor equation is marginally more reliable than the Henry equation for prediction of RMR at the individual level. This is approximately agreeable with a study involving 239 US adults, which showed that the Henry equation predicted RMR accurately in 74 % of individuals, whilst the Mifflin–St Jeor equation provided accurate RMR predictions in 79 % of individuals; however, both equations performed poorer in a Dutch cohort^(13)^.

In the present analysis, all equations except Schofield demonstrated a tendency to systematically over- or underestimate RMR in individuals with relatively higher or lower RMR, which is in line with findings reported elsewhere^(46)^. This has implications for the lightest- and heaviest-weighing subjects in this sample, and therefore, is likely to affect those with low and high BMI. The number of underweight participants in this analysis was modest though prediction accuracy was low in underweight women for all equations, with the Harris–Benedict equation being particularly susceptible to error. Systematic overestimation of RMR was found in women with low RMR and thus most likely in those with low BMI, which would suggest that overestimation rather than underestimation of RMR can expected in underweight women. Although our findings should be interpreted with caution given the small numbers of underweight subjects, a study containing a larger underweight population showed even poorer prediction accuracy than in our dataset^(47)^. This might be because fewer underweight individuals participated in the original development and validation of equations, or that anthropometric measurements and demographic characteristics are unable to predict the majority of variance in RMR measurements in this group.

In the regression analyses, very little of the prediction error was explained by age, height or BMI. This may partly be explained by factors not investigated within this analysis which influence RMR, including body temperature^(47)^, sympathetic nervous system activity^(48)^, circadian and menstrual rhythms^(49)^, thyroid function^(5)^, ethnicity^(50)^ and genetic factors^(51,52)^. These factors are also unable to be factored into prediction equations, and this underlies their inaccuracy at the individual level for a proportion of individuals.

A strength of the present study was that we had data available for large numbers of females across BMI categories, meaning we generalise findings to women though we make no assumptions regarding accuracy of these equations in males. Although all prediction equations have been derived from healthy populations, they are commonly applied in hospital settings where energy requirements of patients are altered by physiological stress imposed by disease state and factors such as surgery, infection, inflammation and changes in body composition^(22,49)^. However, it should be appreciated that this evaluation was conducted in healthy women and overgeneralising to other populations should be avoided. Predictions of RMR and energy requirements of individuals incur errors using all equations, so careful monitoring of body-weight changes remains paramount to assess whether nutritional targets are being met^(49)^. It has been reported that validity of RMR prediction equations can be influenced by the accuracy of the reference method (indirect calorimetry), with some metabolic carts having insufficient accuracy^(53)^ and that RMR is not necessarily concordant between instruments^(54)^. However, in the present study weekly alcohol burning tests, conducted on Oxycon Pro produced CV of ≤1⋅7 and Deltatrac is considered to exhibit the greatest accuracy of all metabolic carts. Furthermore, in regression analyses, accounting for the confounding effect of BMI and age, no difference was found in absolute or percentage prediction error between the two devices for obtaining RMR.

There are some limitations to this analysis. For reasons of feasibility and generalisability, we evaluated RMR prediction equations which used only routine anthropometric variables. Aggregate predictions of RMR, incorporating numerous equations, have been shown to significantly improve accuracy and reduce potential for selecting the worst-performing equation for an individual^(55)^. A further alternative might be to develop BMI-specific prediction equations, which were superior when compared with generic equations in a large German population, particularly in underweight individuals, with no additional benefit of including FFM in equation design^(14)^. Incorporating additional, simple anthropometric variables (for example, waist and hip circumference) within prediction equations may also improve accuracy^(56)^. More affordable hand-held calorimeters are becoming available and may provide a more accurate alternative to prediction equations^(57)^, though others have reported that these machines have limited utility and traditional metabolic carts should remain the preferred method for estimating nutritional requirements where feasible^(58)^.

In conclusion, the equation of Mifflin–St Jeor is the most reliable for predicting RMR, closely followed by the Henry equation, though clinically relevant errors can still be expected in about one-third of individuals, most commonly in individuals with obesity, or who are underweight. Although there is room for improvement, prediction equations do continue to provide convenient estimations of RMR and a starting point for estimating daily energy requirements and developing dietary interventions.

## References

[1] World Health Organization (1985) Energy and Protein Requirements. Report of a Joint FAO/WHO/UNU Expert Consultation, World Health Organization Technical Report Series no. 724. Geneva: WHO. 3937340

[2] Frankenfield D, Roth-Yousey L, Compher C, (2005) Comparison of predictive equations for resting metabolic rate in healthy nonobese and obese adults: a systematic review. J Am Diet Assoc 105, 775–789. 15883556 10.1016/j.jada.2005.02.005

[3] Ravussin E, Lillioja S, Anderson TE, (1986) Determinants of 24-hour energy-expenditure in man. Methods and results using a respiratory chamber. J Clin Invest 78, 1568–1578. 3782471 10.1172/JCI112749 PMC423919

[4] Javed F, He Q, Davidson LE, (2010) Brain and high metabolic rate organ mass: contributions to resting energy expenditure beyond fat-free mass. Am J Clin Nutr 91, 907–912. 20164308 10.3945/ajcn.2009.28512 PMC2844678

[5] Muller MJ, Bosy-Westphal A, Kutzner D, (2002) Metabolically active components of fat-free mass and resting energy expenditure in humans: recent lessons from imaging technologies. Obes Rev 3, 113–122. 12120418 10.1046/j.1467-789x.2002.00057.x

[6] Sabounchi NS, Rahmandad H & Ammerman A (2013) Best-fitting prediction equations for basal metabolic rate: informing obesity interventions in diverse populations. Int J Obes 37, 1364–1370. 10.1038/ijo.2012.218 PMC4278349 23318720

[7] Schofield WN (1985) Predicting basal metabolic rate, new standards and review of previous work. Hum Nutr Clin Nutr 39, 5–41. 4044297

[8] Henry CJK (2005) Basal metabolic rate studies in humans: measurement and development of new equations. Public Health Nutr 8, 1133–1152. 16277825 10.1079/phn2005801

[9] Mifflin MD, St Jeor ST, Hill LA, (1990) A new predictive equation for resting energy expenditure in healthy individuals. Am J Clin Nutr 51, 241–247. 2305711 10.1093/ajcn/51.2.241

[10] Harris JA & Benedict FG (1918) A biometric study of human basal metabolism. Proc Natl Acad Sci U S A 4, 370–373. 16576330 10.1073/pnas.4.12.370 PMC1091498

[11] Owen OE, Kavle E, Owen RS, (1986) A reappraisal of caloric requirements in healthy women. Am J Clin Nutr 44, 1–19. 3728346 10.1093/ajcn/44.1.1

[12] Owen OE, Holup JL, Dalessio DA, (1987) A reappraisal of the caloric requirements of men. Am J Clin Nutr 46, 875–885. 3687821 10.1093/ajcn/46.6.875

[13] Weijs PJM, Kruizenga HM, van Dijk AE, (2008) Validation of predictive equations for resting energy expenditure in adult outpatients and inpatients. Clin Nutr 27, 150–157. 17961867 10.1016/j.clnu.2007.09.001

[14] Mueller MJ, Bosy-Westphal A, Klaus S, (2004) World Health Organization equations have shortcomings for predicting resting energy expenditure in persons from a modern, affluent population: generation of a new reference standard from a retrospective analysis of a German database of resting energy expenditure. Am J Clin Nutr 80, 1379–1390. 15531690 10.1093/ajcn/80.5.1379

[15] 15. Weijs PJM & Vansant GAAM (2010) Validity of predictive equations for resting energy expenditure in Belgian normal weight to morbid obese women. Clin Nutr 29, 347–351. 19853980 10.1016/j.clnu.2009.09.009

[16] Maraki MI, Panagiotakos DB, Jansen LT, (2018) Validity of predictive equations for resting energy expenditure in Greek adults. Ann Nutr Metab 72, 134–141. 29393125 10.1159/000486320

[17] Ruiz JR, Ortega FB, Rodriguez G, (2011) Validity of resting energy expenditure predictive equations before and after an energy-restricted diet intervention in obese women. PLoS ONE 6, e23759. 21909404 10.1371/journal.pone.0023759 PMC3167807

[18] Flack KD, Siders WA, Johnson L, (2016) Cross-validation of resting metabolic rate prediction equations. J Acad Nutr Diet 116, 1413–1422. 27138231 10.1016/j.jand.2016.03.018

[19] Frankenfield DC, Rowe WA, Smith JS, (2003) Validation of several established equations for resting metabolic rate in obese and nonobese people. J Am Diet Assoc 103, 1152–1159. 12963943 10.1016/s0002-8223(03)00982-9

[20] Frankenfield DC (2013) Bias and accuracy of resting metabolic rate equations in non-obese and obese adults. Clin Nutr 32, 976–982. 23631843 10.1016/j.clnu.2013.03.022

[21] European Food Safety Authority (EFSA) Panel on Dietetic Products, Nutrition and Allergies (NDA) (2013) Scientific opinion on dietary reference values for energy. EFSA J 11, 3005. 10.2903/j.efsa.2013.3005 PMC13159830 42124964

[22] Todorovic VE & Micklewright A (2011) A Pocket Guide to Clinical Nutrition, 4th ed. Birmingham: PENG, The Parenteral and Enteral Nutrition Group of the British Dietetic Association.

[23] Scientific Advisory Committee on Nutrition (2012) Dietary Reference Values for Energy. London: The Stationery Office.

[24] Alfheeaid H, Gerasimidis K, Nastase A-M, (2018) Impact of phenylketonuria type meal on appetite, thermic effect of feeding and postprandial fat oxidation. Clin Nutr 37, 851–857. 28318688 10.1016/j.clnu.2017.03.005

[25] Manthou E, Gill JMR, Wright A, (2010) Behavioral compensatory adjustments to exercise training in overweight women. Med Sci Sports Exercise 42, 1221–1228. 10.1249/MSS.0b013e3181c524b7 19997033

[26] Manthou E, Gill JMR & Malkova D (2015) Effect of exercise programs with aerobic exercise sessions of similar intensity but different frequency and duration on health-related measures in overweight women. J Phys Act Health 12, 80–86. 24896583 10.1123/jpah.2013-0047

[27] Fatima S, Gerasimidis K, Wright C, (2015) Response of appetite and potential appetite regulators following intake of high energy nutritional supplements. Appetite 95, 36–43. 26119811 10.1016/j.appet.2015.06.010

[28] Barwell ND, Malkova D, Leggate M, (2009) Individual responsiveness to exercise-induced fat loss is associated with change in resting substrate utilization. Metabolism 58, 1320–1328. 19501861 10.1016/j.metabol.2009.04.016 PMC2731848

[29] Thom G, Dombrowski SU, Brosnahan N, (2020) The role of appetite-related hormones, adaptive thermogenesis, perceived hunger and stress in long-term weight-loss maintenance: a mixed-methods study. Eur J Clin Nutr 74, 622–632. 32020057 10.1038/s41430-020-0568-9

[30] Compher C, Frankenfield D, Keim N, (2006) Best practice methods to apply to measurement of resting metabolic rate in adults: a systematic review. J Am Diet Assoc 106, 881–903. 16720129 10.1016/j.jada.2006.02.009

[31] Frayn KN (1983) Calculation of substrate oxidation rates *in vivo* from gaseous exchange. J Appl Physiol 55, 628–634. 6618956 10.1152/jappl.1983.55.2.628

[32] Bland JM & Altman DG (1986) Statistical methods for assessing agreement between two methods of clinical measurement. Lancet i, 307–310. 2868172

[33] Madden AM, Mulrooney HM & Shah S (2016) Estimation of energy expenditure using prediction equations in overweight and obese adults: a systematic review. J Hum Nutr Diet 29, 458–476. 26923904 10.1111/jhn.12355

[34] Carpenter A, Pencharz P & Mouzaki M (2015) Accurate estimation of energy requirements of young patients. J Pediatric Gastroentr Nutr 60, 4–10. 10.1097/MPG.0000000000000572 25238120

[35] Miller S, Milliron B-J & Woolf K (2013) Common prediction equations overestimate measured resting metabolic rate in young hispanic women. Top Clin Nutr 28, 120–135. 24058263 10.1097/TIN.0b013e31828d7a1b PMC3779143

[36] Dobratz JR, Sibley SD, Beckman TR, (2007) Predicting energy expenditure in extremely obese women. J Parenter Enteral Nutr 31, 217–227. 10.1177/0148607107031003217 17463148

[37] Berger MM & Pichard C (2012) Best timing for energy provision during critical illness. Crit Care 16, 215. 22429787 10.1186/cc11229 PMC3681360

[38] Villet S, Chiolero RL, Bollmann MD, (2005) Negative impact of hypocaloric feeding and energy balance on clinical outcome in ICU patients. Clin Nutr 24, 502–509. 15899538 10.1016/j.clnu.2005.03.006

[39] Singer P, Anbar R, Cohen J, (2011) The Tight Calorie Control Study (TICACOS): a prospective, randomized, controlled pilot study of nutritional support in critically ill patients. Intensive Care Med 37, 601–609. 21340655 10.1007/s00134-011-2146-z

[40] McClave SA, Lowen CC, Kleber MJ, (1998) Are patients fed appropriately according to their caloric requirements? J Parenter Enteral Nutr 22, 375–381. 10.1177/0148607198022006375 9829611

[41] Grau T, Bonet A, Rubio M, (2007) Liver dysfunction associated with artificial nutrition in critically ill patients. Crit Care 11, R10. 17254321 10.1186/cc5670 PMC2147066

[42] Dvir D, Cohen J & Singer P (2006) Computerized energy balance and complications in critically ill patients: an observational study. Clin Nutr 25, 37–44. 16321459 10.1016/j.clnu.2005.10.010

[43] Frost G, Masters K, King C, (1991) A new method of energy prescription to improve weight loss. J Hum Nutr Diet 4, 369–373. 10.1111/j.1365-277X.2007.00775.x 17539863

[44] Department of Health (1991). Dietary Reference Values for Food Energy and Nutrients for the United Kingdom: Report of the Panel on Dietary Reference Values of the Committee on Medical Aspects of Food Policy. Report on Health and Social Subjects 41. London: The Stationery Office. 1961974

[45] Judges D, Knight A, Graham E, (2012) Estimating energy requirements in hospitalised underweight and obese patients requiring nutritional support: a survey of dietetic practice in the United Kingdom. Eur J Clin Nutr 66, 394–398. 22190132 10.1038/ejcn.2011.211

[46] Jesus P, Achamrah N, Grigioni S, (2015) Validity of predictive equations for resting energy expenditure according to the body mass index in a population of 1726 patients followed in a Nutrition Unit. Clin Nutr 34, 529–535. 25016971 10.1016/j.clnu.2014.06.009

[47] Rising R, Keys A, Ravussin E, (1992) Concomitant interindividual variation in body temperature and metabolic rate. Am J Physiol 263, 730–734. 10.1152/ajpendo.1992.263.4.E730 1415692

[48] Spraul M, Ravussin E, Fontvieille AM, (1993) Reduced sympathetic nervous activity – a potential mechanism predisposing to body-weight gain. J Clin Invest 92, 1730–1735. 8408625 10.1172/JCI116760 PMC288333

[49] Weekes CE (2007) Controversies in the determination of energy requirements. Proc Nutr Soc 66, 367–377. 17637089 10.1017/S0029665107005630

[50] Sharp TA, Bell ML, Grunwald GK, (2002) Differences in resting metabolic rate between white and African-American young adults. Obes Res 10, 726–732. 12181380 10.1038/oby.2002.99

[51] Konarzewski M & Ksiazek A (2013) Determinants of intra-specific variation in basal metabolic rate. J Comp Physiol B 183, 27–41. 22847501 10.1007/s00360-012-0698-z PMC3536993

[52] Bogardus C, Lillioja S, Ravussin E, (1986) Familial dependence of the resting metabolic rate. N Engl J Med 315, 96–100. 3724804 10.1056/NEJM198607103150205

[53] Galgani JE, Castro-Sepulveda M, Perez-Luco C, (2018) Validity of predictive equations for resting metabolic rate in healthy humans. Clin Sci 132, 1741–1751. 29967004 10.1042/CS20180317

[54] Schadewaldt P, Nowotny B, Strassburger K, (2013) Indirect calorimetry in humans: a postcalorimetric evaluation procedure for correction of metabolic monitor variability. Am J Clin Nutr 97, 763–773. 23446893 10.3945/ajcn.112.035014

[55] Wells JCK, Williams JE, Haroun D, (2009) Aggregate predictions improve accuracy when calculating metabolic variables used to guide treatment. Am J Clin Nutr 89, 491–499. 19141697 10.3945/ajcn.2008.26629

[56] Hedayati KK & Dittmar M (2011) Body circumferences are predictors of weight adjusted resting energy expenditure in older people. J Nutr Health Aging 15, 803–808.2215976510.1007/s12603-011-0072-y

[57] Hipskind P, Glass C, Charlton D, (2011) Do handheld calorimeters have a role in assessment of nutrition needs in hospitalized patients? A systematic review of literature. Nutr Clin Pract 26, 426–433. 21775638 10.1177/0884533611411272 PMC4581882

[58] Anderson EJ, Sylvia LG, Lynch M, (2014) Comparison of energy assessment methods in overweight individuals. J Acad Nutr Diet 114, 273–278. 24051107 10.1016/j.jand.2013.07.008 PMC4350191

