# Validity of predictive equations to estimate RMR in females with varying BMI - CORRIGENDUM

**DOI:** 10.1017/jns.2020.20

**Published:** 2020-06-18

**Authors:** George Thom, Konstantinos Gerasimidis, Eleni Rizou, Hani Alfheeaid, Nick Barwell, Eirini Manthou, Sadia Fatima, Jason M. R. Gill, Michael E. J. Lean, Dalia Malkova

**Affiliations:** 1 Human Nutrition, School of Medicine, College of Medical, Veterinary and Life Sciences, University of Glasgow, New Lister Building, Glasgow Royal Infirmary, Glasgow G31 2ER, UK; 2 Department of Food Science & Human Nutrition, College of Agriculture & Veterinary Medicine, Qassim University, Buraydah City, P. C. 51452, Saudi Arabia; 3 BHF Glasgow Cardiovascular Research Centre, Institute of Cardiovascular and Medical Sciences, College of Medical, Veterinary and Life Sciences, University of Glasgow, Glasgow G12 8TA, UK

In the aforementioned article there was an incorrect affiliation published for the fourth author; this has since been rectified. The authors apologise for this error.
